# Creatine Supplementation Potentiates Exercise Protective Effects against Doxorubicin-Induced Hepatotoxicity in Mice

**DOI:** 10.3390/antiox12040823

**Published:** 2023-03-28

**Authors:** Loriane R. L. Costa Godinho, Paola S. Cella, Tatiana A. S. Guimarães, Guilherme H. Dantas Palma, Jonathan H. C. Nunes, Rafael Deminice

**Affiliations:** Physical Education and Sports Institute, State University of Londrina, Londrina 86057-970, PR, Brazil

**Keywords:** chemotherapy, liver toxicity, strength training, food supplement, cancer

## Abstract

We tested the hypothesis that creatine supplementation may potentiate exercise’s protective effects against doxorubicin-induced hepatotoxicity. Thirty-eight Swiss mice were randomly allocated into five groups: control (C, *n =* 7), exercised (Ex, *n =* 7), treated with doxorubicin (Dox, *n =* 8), treated with doxorubicin and exercised (DoxEx, *n =* 8), and treated with doxorubicin, exercised, and supplemented with creatine (DoxExCr, *n =* 8). Doxorubicin was administered weekly (i.p.) for a total dose of 12 mg/kg. Creatine supplementation (2% added to the diet) and strength training (climbing stairs, 3 times a week) were performed for a total of 5 weeks. The results demonstrated that doxorubicin caused hepatotoxicity, which was evidenced by increased (*p* < 0.05) hepatic markers of inflammation (i.e., TNF-α and IL-6) and oxidative damage, while the redox status (GSH/GSSG) was reduced. The plasma concentrations of liver transaminases were also significantly (*p* < 0.05) elevated. Furthermore, doxorubicin-treated animals presented hepatic fibrosis and histopathological alterations such as cellular degeneration and the infiltration of interstitial inflammatory cells. Exercise alone partly prevented doxorubicin-induced hepatotoxicity; thus, when combined with creatine supplementation, exercise was able to attenuate inflammation and oxidative stress, morphological alterations, and fibrosis. In conclusion, creatine supplementation potentiates the protective effects of exercise against doxorubicin-induced hepatotoxicity in mice.

## 1. Introduction

Anthracyclines such as doxorubicin are widely used in cancer treatment due to their potent antineoplastic effects against solid tumors, lymphoma, and leukemia [1]. Although effective against tumor tissues, doxorubicin is toxic for healthy tissues; as previously studies have demonstrated, doxorubicin promotes oxidative stress and inflammation in both cardiac and skeletal muscles, the liver, kidneys and others [2,3,4,5]. Among all these adverse effects, liver toxicity has a significant impact on cancer treatment and may develop into liver dysfunction, which, in the worst-case scenario, causes death. In cases where the liver’s injury is reversible, antineoplastic treatment may be suspended, although doing so will delay cancer treatment. Thus, new strategies that can protect the liver from chemotherapy agent-induced toxicity are needed to increase the tolerance and effectiveness of cancer treatment and thereby improve the quality of life of cancer patients. Although their mechanisms remain poorly elucidated, reactive oxygen species (ROS) production and oxidative stress are considered to be the key triggers of doxorubicin-induced liver damage [6]. Indeed, studies have demonstrated that doxorubicin treatment promotes reduced levels of hepatic antioxidant enzymes, apoptosis, inflammation, and mitochondrial dysfunction [7,8,9,10].

Regular physical exercise is associated with better health and decreased overall mortality [11,12]. Recently, the positions and consensuses from scientific and medical entities have proposed physical exercise, including strength training (ST), as part of cancer treatment during chemotherapy [13,14]. ST has been found to be safe and to improve muscle mass and strength in patients with cancer undergoing neoadjuvant and adjuvant therapy [15]. Indeed, physically active patients with cancer have a lower risk of mortality than inactive ones [16]. Regarding liver health, it was recently shown that aerobic exercise preconditioning can sufficiently prevent doxorubicin-induced liver toxicity [10,17,18]. However, whether ST is tolerable and effective in protecting the liver during chemotherapy with doxorubicin remains unknown.

Creatine, a compound widely used is sports due its ergogenic effects, has gained attention from the medical field in recent years due to its protective effects in various situations [19,20]. Most of creatine’s muscle-protective effects are attributed to its mild antioxidant and anti-inflammatory activity, properties that were recently demonstrated in hepatic-damaging situations [15,21,22]. Beyond that, studies from our group and others have shown that creatine prevents cancer-induced hepatotoxicity [21], hepatic damage induced by a high-fat diet, and fatty liver disease [22,23]. Recently, Aljobaily et al. [24] found that creatine supplementation also alleviates liver fibrosis, inflammation, oxidative stress, and cellular senescence induced by a bolus injection of doxorubicin of 15 mg/kg. Although it is important, past studies from the literature have not reproduced the characteristics of doxorubicin treatment in humans who receive doxorubicin intermittently with rest periods. In addition, the effects of ST and creatine supplementation on doxorubicin-induced hepatic injury is unknown. To investigate the effects of ST and creatine supplementation of liver hepatotoxicity is relevant, given that both have been demonstrated to protect against strength loss and muscle atrophy imposed by cancer and its treatment [15,25,26,27], all capacities that are strictly associated with mortality in cancer patients [28].

Thus, the aim of our study was to investigate the effects of ST, combined with or without creatine supplementation on doxorubicin-induced hepatotoxicity. We hypothesized that creatine supplementation may potentiate exercise’s protective effects against hepatotoxicity induced by doxorubicin treatment. To test that hypothesis, we proposed a doxorubicin injection protocol that mimics regular chemotherapy treatment in humans, with the doxorubicin injection interspersed with rest periods. That strategy also allowed us to incorporate ST during the doxorubicin treatment.

## 2. Materials and Methods

### 2.1. Animals and Study Design

Thirty-eight male Swiss mice that were 6–8 weeks old were obtained from the facilities of the State University of Londrina’s Animal Care Unit. Animals were individually housed and maintained on a 12 h light–dark cycle at a mean temperature of 22 °C, with free access to food and water for the entirety of the experimental period. All procedures were approved by the Ethics Committee for Animal Use at the same institution (#11131.2019.07) and complied with the ethical standards of the Brazilian College of Animal Experimentation and of the Declaration of Helsinki and its amendments.

Animals were randomly assigned to five groups: control (C, *n =* 7), exercised (Ex, *n =* 7), treated with doxorubicin (Dox, *n =* 8), treated with doxorubicin and exercised (DoxEx, *n =* 8), and treated with doxorubicin, exercised, and supplemented with creatine (DoxExCr, *n =* 8). Doxorubicin hydrochloride Bergamo^®^ (Taboão da Serra, São Paulo, Brazil) was intraperitoneally injected once a week for 5 weeks for a total dosage of 12 mg/kg. All groups received an AIN-93G; the DoxExCr group received same diet supplemented with 2% creatine monohydrate, which was manipulated, formulated, and commercialized by Rhoster^®^ (Araçoiaba da Serra, São Paulo, Brazil). (Sigma^®^, St. Louis, MO, USA). Supplementation with 2% creatine was chosen based on previous studies demonstrating that it increased plasma and hepatic creatine concentrations [29]. The creatine supplementation started 1 week before the first doxorubicin injection and the beginning of exercise training and continued throughout the 6-week experimental period. Body weight was assessed daily and diet consumption was assessed twice a week for 5 weeks. Animals were euthanized 48 h after the last training session, which was 6 weeks after the beginning of the experiment.

### 2.2. ST Protocol

ST consisted of a previously described ladder-climbing protocol [25]. The ladder (60 × 18 cm, 0.8 cm grid, 90° incline) was projected so animals could perform 8–12 dynamic movements per climb. One week before starting the ST protocols, all mice were familiarized with the exercise apparatus until they voluntarily climbed the ladder without any stimulus. No attached load was used during that period. Briefly, the ST regimen consisted of a maximum of 8 sets of climbing a ladder 3 times a week for 5 weeks, where twice weekly, the animals performed training of 60% of their maximum carrying load capacity, interspersed with one training with their maximum carrying load capacity, which was used to adjust the weekly training load. In the end of the adaptation week, all animals performed the first maximum carrying load test 48 h before the first administration of doxorubicin. The Ex, DoxEx, and DoxExCr groups performed ST for 5 weeks in a total of 15 ST sessions.

The maximum carrying load test consisted of four ladder climbs while carrying 50%, 75%, 90%, and 100% of the animal’s body weight. For the fifth ladder climb, an additional 3 g was added to the load. That procedure was repeated until the mice could no longer completely climb the ladder due to the weight in three consecutive attempts. The load carried in the last successful climbing attempt was used as the maximum carrying load capacity. By contrast, the mice from the C and Dox groups remained sedentary during the entire period of the experimental protocol.

Strength gain was determined by the difference between last and first maximal strength tests. Total workload was determined by multiplying the number of completed sets per carried load in each training session.

### 2.3. Euthanasia and Tissue Preparation

Next, 48 h after the last training session and after 6 h of fasting, the mice were anesthetized by inhalation with isoflurane (5%) and euthanized by exsanguination. Euthanasia was performed between 9:00 a.m. and 1 p.m. Blood was collected into heparinized tubes and centrifuged at 1000× *g* for 15 min at 4 °C, after which, the plasma was stored at –80 °C for further analysis. The liver was excised and weighed; a portion of the liver was stored at −80 °C until analysis, while another portion was prepared for histological analysis.

### 2.4. Histological Analysis

For histological analysis, liver fragments from the right lobe were placed in a 4% formalin solution for 24 h. After fixation, the samples were dehydrated in increasing concentrations of ethanol (i.e., 70, 95, and 100%), followed by two xylene changes. After that, the fixed and dehydrated liver fragment were placed in paraffin, and the pieces were cut into 5 μm portions and mounted onto glass slides. Before staining, the slices underwent deparaffinization and were later stained with hematoxylin and eosin (H&E). Next, histopathological analysis was performed blindly by an experienced pathologist, using approximately 35 representative images of each group that were digitally acquired with a light microscope (Biopta^®^) with an attached camera (AmScope^®^), and they were analyzed under 20× magnification. Tissue sections were analyzed to assess the occurrence of cellular degeneration, the infiltration of interstitial inflammatory cells, necrotic zones, and the alteration of tissue organization [30]. First, to determine the severity of cell degeneration, the number of cells that demonstrated any changes (i.e., dilation, vacuolization, and pyknotic nuclei) was determined visually and classified as grade 0 (i.e., no change from normal histology); grade 1 (i.e., limited number of isolated cells representing ≤ 5% of the total number of cells); grade 2 (i.e., groups of cells representing 5–30% of the total number of cells); or grade 3 (i.e., diffuse cell damage representing > 30% of the total number of cells). Second, inflammatory activity was classified as grade 0 (i.e., no cell infiltration); grade 1 (i.e., mild leukocyte infiltration in 1–3 cells per visual field); grade 2 (i.e., moderate infiltration in 4–6 leukocytes per visual field); or grade 3 (i.e., intense infiltration by neutrophils representing > 6 leukocytes per visual field). Third, the level of necrosis was classified as grade 0 (i.e., no necrosis); grade 1 (i.e., scattered necrotic foci); grade 2 (i.e., confluent necrotic areas); or grade 3 (i.e., massive areas of necrosis). Fourth and finally, the severity of tissue disorganization was classified as 0 (i.e., normal structure); 1 (i.e., less than one-third of the tissue); 2 (i.e., more than one-third but less than two-thirds of the tissue); or 3 (i.e., more than two-thirds of the tissue).

The accumulation of fibrous tissue was evaluated according to approximately 30 representative images of each group stained with picrosirius red (20× magnification) that were digitally acquired under a light microscope (Biopta^®^) with an attached camera (AmScope^®^) and analyzed by a blinded observer using Image J software (National Institute of Health, Bethesda, MD, USA). The area of liver tissue occupied by collagen (i.e., stained red) was quantified for each visual field and expressed as a percentage (%) of the total field. Multiple fields per liver were evaluated to ensure that the data were representative.

### 2.5. Interleukins, Oxidative Stress, and Oxidative Damage Analysis

Frozen liver samples were homogenized in ice-cold phosphate-buffered saline. Reduced glutathione (GSH) and oxidized glutathione (GSSG) hepatic concentrations were determined using the method by Rahman et al. [31]. To evaluate oxidant damage, the supernatants were used for the liver concentration of malondialdehyde (MDA) as lipid peroxidation parameters following the method by Spirlandeli et al. [32]. Advanced oxidation protein products (AOPPs) were also determined in the liver following the method by Witko-Sarsat et al. [33].

The homogenates were also used to determine tumor necrosis factor alpha (TNF-α, Ref. No. 88-7324-88) and interleukin 6 (IL-6, Ref. No. 88-7064-88) hepatic concentration using commercially available ELISA kits (Thermo Fisher Scientific^®^, Waltham, MA, USA).

The plasma activity of aspartate aminotransferase (AST, Ref. No. 109-4/30) and alanine transaminase (ALT, Ref. No. 108-2/100) levels were determined spectrophotometrically using a commercially available kit from Labtest Diagnóstica^®^ (Lagoa Santa, MG, Brazil).

### 2.6. Statistical Analyses

Data were analyzed using GraphPad Prism 7. The Shapiro–Wilk test was used to analyze the normality of data distribution; data that assumed normal distribution were recorded as means with standard deviations. To compare the parametric data and identify possible between-group differences, a two-way analysis of variance (ANOVA) was conducted, followed by Tukey’s post hoc test. For non-parametric data (i.e., ALT and TNF-α hepatic concentration), the Kruskal–Wallis test with Dunn’s post hoc test was performed. The level of significance was set at *p* < 0.05 in all cases.

## 3. Results

### 3.1. Effects of Creatine and ST on Doxorubicin-Induced Weight Loss and Muscle Strength

**Figure 1:**
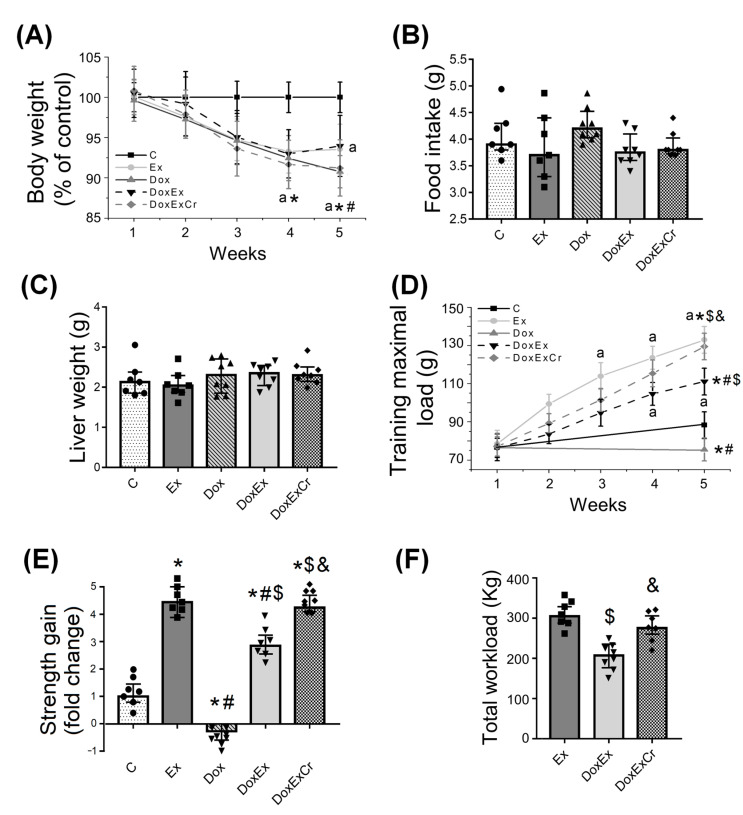
Creatine supplementation potentiates protective effects of ST by enhancing training load capacity and strength gain. Weekly changes in (**A**) total body weight, average (**B**) food intake, and (**C**) liver weight. Changes in (**D**) training maximal load over 5 weeks, (**E**) strength gain, and (**F**) total workload for groups control (C, *n =* 7), exercised (Ex, *n =* 7), treated with doxorubicin (Dox, *n =* 8), treated with doxorubicin and exercised (DoxEx, *n =* 8), and treated with doxorubicin, exercised, and supplemented with creatine (DoxExCr, *n =* 8). Data are expressed as mean ± SD. Between-group differences were assessed by two-way ANOVA using Tukey’s post hoc test with *p* < 0.05 considered statistical significance. * indicates a significant difference from control group; # indicates a significant difference from the exercised group; $ indicates a significant difference from the doxorubicin-treated group; and & indicates a significant difference from the doxorubicin-treated exercised group. ^a^ Indicates a significant difference from weeks 1.

Five cycles of doxorubicin injection induced progressive body weight loss from the third week to the end of the experiment; doxorubicin caused a total weight loss of 9.3% compared to the control. Although exercised animals tend to regain weight at the end of the injection cycle, neither ST nor ST combined with creatine protected mice from doxorubicin-induced weight loss (total weight loss of 6 and 8.1%, respectively) (Figure 1A). Food intake or liver weight were not changed by doxorubicin injections, ST, or creatine supplementation (Figure 1B,C).

After 5 weeks, doxorubicin-treated mice presented reduced strength, which was clearly demonstrated by their reduced maximal carrying load (Figure 1D), and strength gain (Figure 1E). In the opposite, ST was able to prevent doxorubicin-induced strength loss (Figure 1D,E). Notably, creatine potentiated the protective effects of ST; creatine-supplemented mice presented a higher training maximal load, strength gain, and total workload when compared to ST mice that were not supplemented. Markedly, creatine-supplemented mice muscle strength gain and total workload reached a similar level to that of trained heathy mice (Figure 1D–F).

### 3.2. Effects of Creatine and ST on Doxorubicin-Induced Hepatotoxicity

**Figure 2:**
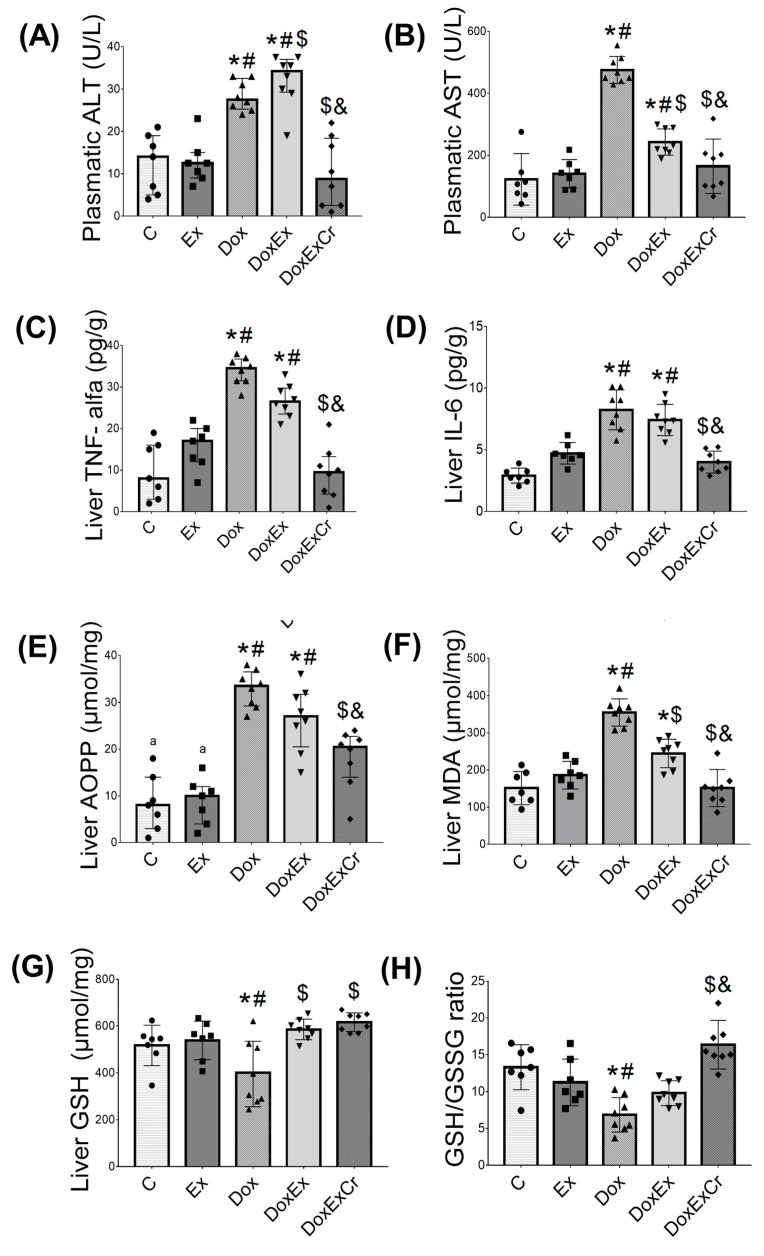
Creatine supplementation associated with ST prevented doxorubicin-induced hepatotoxicity. Plasma concentration of hepatic transaminases, (**A**) ALT and (**B**) AST, hepatic concentration of inflammatory mediators, (**C**) TNF-α and (**D**) IL-6, and oxidative damage and antioxidant defense markers (**E**) AOPP, (**F**), MDA (**G**), and GSH, and (**H**) GSH/GSSG ratio for groups control (C, *n =* 7), exercised (Ex, *n =* 7), treated with doxorubicin (Dox, *n =* 8), treated with doxorubicin and exercised (DoxEx, *n =* 8), and treated with doxorubicin, exercised, and supplemented with creatine (DoxExCr, *n =* 8). Data are expressed as mean ± SD. Differences between groups were surveyed by two-way analysis of variance using Tukey’s post hoc test, with *p* < 0.05 considered statistical significance. * indicates a significant difference from control group; # indicates a significant difference from the exercised group; $ indicates a significant difference from the doxorubicin-treated group; and & indicates a significant difference from the doxorubicin-treated exercised group. ALT, alanine transaminase; AST, aspartate aminotransferase; TNF-α, tumor necrosis factor-alpha; IL-6, interleukin-6; AOPP, advanced oxidation protein products; MDA, malondialdehyde; GSH, reduced glutathione; GSH/GSSG, and reduced glutathione-to-oxidized glutathione ratio.

Biochemical data suggest that the five cycles of doxorubicin treatment promoted hepatotoxicity compared with the control group, as evidenced by the significantly (*p* < 0.05) increased plasma ALT and AST activity (Figure 2A,B) and elevated hepatic inflammatory mediators TNF-α and IL-6 (Figure 2C,D). In addition, doxorubicin promoted liver oxidative stress demonstrated by elevated AOPPs and MDA (Figure 2E,F), and a reduced redox status evidenced by the GSH/GSSG ratio (Figure 2G,H), all compared with the control. By contrast, ST partly protected the liver from doxorubicin-induced hepatotoxicity by slightly decreasing the AST plasma concentration (Figure 2B) and hepatic oxidative damage, as demonstrated by the significant reduction in AOPPs and MDA (Figure 2E,F). However, ST neither attenuated the elevated hepatic concentration of TNF-α or IL-6 (Figure 2C,D) nor completely restored liver redox status, as also demonstrated by the GSH/GSSG ratio (Figure 2G).

When associated with creatine supplementation, ST prevented doxorubicin-induced hepatotoxicity, as indicated by the significant reductions (*p* < 0.05) in ALT and AST plasma activity, the inflammatory mediators TNF-alpha and IL-6, and AOPPs and MDA as oxidative damage markers. Furthermore, hepatic redox status was restored to the control level as demonstrated by an elevated GSH/GSSG ratio. Importantly, ST without doxorubicin treatment did not disrupt hepatic transaminases, inflammatory mediators, or oxidative stress (Figure 2).

### 3.3. Histopathological Analyzes

**Figure 3:**
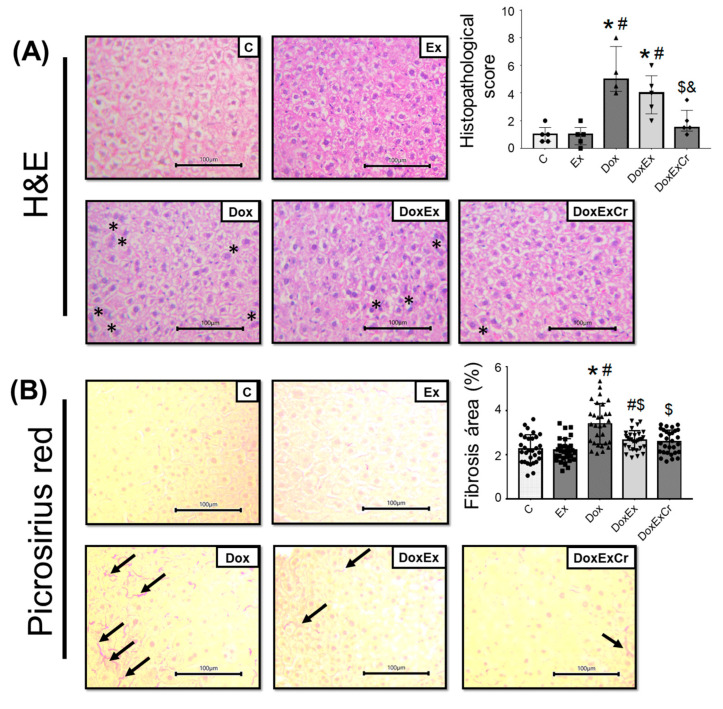
Creatine potentiates ST protection against doxorubicin-induced histopathological alterations. Asterisk indicates cellular degeneration and arrows indicates fibrosis and necrotic areas. Representative (**A**) H&E-stained liver sections and histopathological scores (i.e., cellular degeneration, infiltrate of interstitial inflammatory cells, necrotic zones, and alteration of tissue organization). Representative (**B**) picrosirius red-stained liver sections and percentage of hepatic fibrosis-stained area for groups control (C, *n =* 6), exercised (Ex, *n =* 6), treated with doxorubicin (Dox, *n =* 6), treated with doxorubicin and exercised (DoxEx, *n =* 6), and treated with doxorubicin, exercised, and supplemented with creatine (DoxExCr, *n =* 6). Values are median and interquartile intervals. Differences between the groups were surveyed by *Kruskal–Wallis* using Dun’s post hoc test. Illustrative panels show representative photomicrographs taken under 20× magnification. * indicates a significant difference from control group; # indicates a significant difference from the exercised group; $ indicates a significant difference from the doxorubicin-treated group; and & indicates a significant difference from the doxorubicin-treated exercised group. H&E, hematoxycillin and eosin.

Histopathological evaluation demonstrated that five cycles of doxorubicin injection promoted morphological alterations that were characterized by cellular degeneration, the infiltration of interstitial inflammatory cells, necrotic zones, and tissue disorganization compared with the control group, as evidenced by significantly (*p* < 0.05) elevated histopathological scores compared with the control group (Figure 3A). Although ST did not provide significant protection against doxorubicin-induced histopathological alterations, the combination of creatine with ST was able to significantly (*p* < 0.05) reduce histopathological scores, thereby demonstrating less infiltration of the interstitial inflammatory cells, fewer necrotic zones, and less tissue disorganization (Figure 3A).

Altogether, whether combined with creatine supplementation or not, ST attenuated doxorubicin-increased collagen deposition, as evaluated in the picrosirius red-stained sections (Figure 3B). Moreover, ST without doxorubicin treatment did not cause any significant morphological alterations or fibrosis.

## 4. Discussion

Using a doxorubicin treatment model approximate to that followed for cancer patients, we investigated the effects of ST combined with or without creatine supplementation on doxorubicin-induced hepatotoxicity. Firstly among our major findings, five cycles of weekly doxorubicin injections promoted hepatotoxicity characterized by the increased activity of plasma transaminases and elevated hepatic inflammatory mediators and oxidative stress, which were all accompanied by histopathological disturbances such as cellular degeneration, interstitial inflammatory cell infiltration, and mild fibrosis. Secondly, we demonstrated that ST is tolerable and feasible during doxorubicin injection cycles and it can protect against doxorubicin-induced strength loss, which is compatible with previously studies. Thirdly, ST partly protected the liver against doxorubicin-induced hepatotoxicity by attenuating oxidative damage and hepatic collagen deposition; however, this did not prevent elevated hepatic inflammatory mediators and histopathological alterations. Fourth, creatine potentiated the protective effects of ST, thereby preventing elevated plasma transaminases, hepatic oxidative stress, and inflammatory mediators, as well as reduced liver collagen deposition and tissue disturbances. Finally, our data also confirmed previously data suggesting that ST protects against doxorubicin-induced strength loss and that creatine supplementation potentiated this protective effect. These results confirm our primary hypothesis that creatine supplementation may potentiate exercise’s protective effects against hepatotoxicity induced by doxorubicin treatment.

To the best of our knowledge, our study is the first to demonstrate that ST and creatine supplementation synergistically protect doxorubicin-induced liver toxicity. Those findings are relevant because physical exercise and creatine supplementation are easy and relatively low-cost strategies that can be feasibly incorporated into the chemotherapy routines of patients with cancer. Our results are also significant given the lack of effective treatments for preventing or attenuating liver toxicity induced by doxorubicin. Decreasing liver toxicity during chemotherapy is important because severe doxorubicin-induced hepatotoxicity may interrupt antineoplastic treatment and consequently, this may delay cancer treatment.

Although studies have already demonstrated the protective effects of physical exercise against doxorubicin-induced liver toxicity, most of them have used a bolus injection of doxorubicin to induce liver toxicity [10,17,18]. Considering the fact that most the human’s treatment with chemotherapy using anthracyclines drugs are performed in cycles, we used an experimental approach that mimics human’s chemotherapy treatment. The time among the anthracycline injections (cycles) permits the patient to recover from cumulative toxicity, reducing the side effects and improving the patient’s quality of life; at same time, this is an effective method to promote tumor reduction. The cycles of weekly doxorubicin injection for a total period of 5 weeks that we used generated characteristics close to that seem in humans, such as body weight loss, physical dysfunction, and liver toxicity [10,17,18,34], without causing deaths, which permitted animals to perform ST.

In addition, all the available studies have tested the effects of aerobic exercise preconditioning against doxorubicin-induced liver toxicity. In fact, the effects of ST on doxorubicin-induced liver toxicity and damage are poorly known. To study the effects of ST during chemotherapy treatment is important because several cancer patients do not have enough time between the cancer diagnostic and the initiation of primary treatment (surgery and radiation of neoadjuvant chemotherapy), so ST can be incorporated as preconditioning. The median time to treatment initiation in the USA is 29 days; some cancer types, therefore, have proportionated less time than that, such as renal, lung, and pancreas (ranging from 2 to 18 days) [35]. In addition, to incorporate ST during one’s chemotherapy routine is important because it has been demonstrated to improve muscle mass, strength, and physical function [15], which are all capacities that be little modified by aerobic exercise and are strictly associated with mortality in patients with cancer [28]. Thus, ST has the potential to concomitantly protect against doxorubicin-induced skeletal muscle function decline and liver toxicity.

Although ST has already been demonstrated to protect against doxorubicin-induced cardio [36] and skeletal muscle dysfunction [37], ours is the first investigation demonstrating STs protective powers against doxorubicin-induced hepatotoxicity. Indeed, ST significantly prevented elevated AST plasma concentrations and hepatic oxidative damage (reduced AOPPs and MDA); however, ST neither attenuated the elevated hepatic concentration of TNF-α or IL-6, nor completely restored liver redox status, as also demonstrated by the GSH/GSSG ratio. Previous studies have demonstrated that aerobic exercise ranging from 1 to 5 weeks before doxorubicin injection promotes important protection against doxorubicin-induced hepatotoxicity [17,18,34,38,39]. Indeed, these studies have argued that reduced ROS production and mitochondrial dysfunction are key events in aerobic exercise-promoted protection against doxorubicin-induced hepatotoxicity [18,38]. Although we did not measure any mitochondrial parameters (which can be considered the major limitation of the present study), it is well known that ST causes mild changes in the mitochondrial function [40,41] or, at least, fewer changes than aerobic exercise, and it promotes a mild protection against ROS production and oxidative stress, as demonstrated in the present study. Moreover, we may speculate that ST promoted some antioxidant protection, which seems to be sufficient to partially, but not fully, protect against the doxorubicin-induced hepatotoxicity.

Because ST is important to protect against the doxorubicin-induced skeletal muscle dysfunction, but it did not fully protect doxorubicin-induced liver damage, we combined ST and creatine supplementation, in an attempt to potentiate the protective effects of ST. We demonstrated the combination of ST and creatine supplementation and thereby promoted full liver protection against doxorubicin-imposed liver toxicity and damage, by preventing elevated plasma transaminases, hepatic oxidative stress, and inflammatory mediators. Notably, the combination of ST and creatine supplementation prevented liver morphological alterations and collagen deposition induced by cycles injection of doxorubicin. Indeed, our data demonstrate that ST and creatine supplementation synergistically protect against doxorubicin-induced strength loss and liver toxicity. Since the first evidence described by Lawler et al. [42] demonstrating creatine’s direct antioxidant effects, several studies have demonstrated that creatine protects against oxidative stress and damage in different situations and tissues [43,44], including the liver [22,24,45]. Furthermore, beyond its direct antioxidant properties, creatine has demonstrated pleiotropic action that differentiates itself from classical antioxidant compounds. Studies have demonstrated that creatine has an important role in improving the cellular energy state (CrP/ATP ratio) at the same time that it protects mitochondria from ROS, which would otherwise cause severe energy failure and cell death [44,45,46,47,48,49], key elements of doxorubicin-induced liver toxicity, and damage. The evidence suggests that creatine activates AMPK, which can promote adaptive responses in cells to overcome oxidative stress [47,50]. Another indication of its antioxidant action may relate to the induction of creatine in the thioredoxin–peroxiredoxin system, which can increase NADPHs resynthesis due to the greater bioavailability of ATP [44,48]. In addition, Araujo et al. [51] demonstrated that creatine supplementation acts in an additive manner to physical training to raise antioxidant enzymes in rat livers.

Although studies have already demonstrated that creatine alone can protect the liver against doxorubicin-induced hepatotoxicity [13], the absence of a creatine-isolated group is arguably a limitation of our study. In fact, studies that have tested the effects of creatine supplementation alone against cycling approach doxorubicin-induced hepatotoxicity are scarce; this may be considered in the future studies. The absence of a tumor growth or cancer model for treatment with doxorubicin can also be considered a limitation, even though no studies on the possible protective effects of exercise and/or creatine supplementation against doxorubicin-induced hepatic or muscle toxicity have used that strategy. All things considered, our results underscore the need for more pre-clinical and clinical studies to establish physiological mechanisms and prescribe physical training and creatine supplementation in order to reduce hepatotoxicity and thereby provide a better therapeutic response and quality of life for patients.

## 5. Conclusions

Our data demonstrated the presence of hepatic inflammation, oxidative stress, hepatic fibrosis, and increased markers of liver damage, which are all induced by doxorubicin cycle administration. Importantly, ST was demonstrated to be tolerable and feasible during doxorubicin injection cycles and to protect against doxorubicin-induced strength loss. However, ST alone can only provide partial protection against doxorubicin-induced hepatotoxicity; by contrast, ST-associated creatine supplementation can fully protect livers from developing doxorubicin-induced inflammation, oxidative stress, and damage.

## Data Availability

Data from the study will be made available upon reasonable request.
